# Trial of Allen vs. Hunter

**Published:** 1855-07

**Authors:** Jas. Taylor


### TRIAL OF ALLEN vs. HUNTER.

This trial, which has for so long a time excited much interest in the profession, is now ended, and we here present a full and accurate report of the same to the public.

To secure a fair and impartial report we employed one of our most reliable reporters in the city, Mr. John D. Caldwell, who has, with great care, taken all the evidence of the case which is of interest or importance. In addition to this, he has embodied all the points of law which appear to have a bearing on the case and which may be of much service, legally, to the profession. The able arguments of the counsel on both sides, are given in as condensed a form as is practicable for a fair understanding of the case. The very able charge of the Judge is also given, merely omitting the direct repetition of testimony, which can be found by reference to the report itself. The testimony which is referred to as contained in the various Dental periodicals and Dental works, is necessarily omitted in the Report. It is, however, so marked that the profession can refer to it without any trouble. The case closes with the verdict of the jury.

The case was managed by some of our ablest lawyers, and the trial, throughout, excited much interest. This interest was very much heightened by the vast amount of scientific matter elicited during the examination of some of the witnesses. In this respect, we cannot but regard this case as of service to the profession; giving the public a far more exalted and correct opinion of Dentistry as a science than heretofore. Frequently, during the trial and since, we heard remarks in relation to the general amount of scientific knowledge exhibited by the profession, which would be complimentary to any profession.

The Report, which we present, embodies, we are satisfied, more practical knowledge on this special department of Mechanical Dentistry than can be had in all the published works extant. It gives the experience of some of our best operators in this mode of practice, and will, we think, do much to correct the errors of some and also aid materially in perfecting the improvement itself.

The analyses, by Drs. Slack and Locke, contain much valuable information: giving the contraction and expansion of the materials used by Delabarre, Hunter, and Allen.

To enable us to give the entire report to the profession as early as practicable, we have had to enlarge the present number of the Register, and also leave out other matter we should like to have published. The Report, however, would not do to divide, or, at least, is far more valuable together.

In consequence of other duties devolving upon Mr. Caldwell at this time, the entire Report could not be had in time for its appearance as early as we desired.

We have been at great expense to lay the whole of this case before the profession; and hence, to somewhat reimburse this outlay, we shall issue several hundred extra copies and supply the profession, in this way, much earlier than in any other method. The price charged is just the same, per single number, as the regular subscription price of the Register, of the size we now present it, which is a fair sample of our future issues. The subscription price, therefore, of the Register, hereafter, will be $3·00 per annum. This is in accordance with the request of the the Mississippi Valley Association of Dental Surgeons and many of the profession. Our regular subscribers, who have paid for the Register, will not be charged extra for this number. Those who have never paid can get the present number by doing so; and those who desire can commence their subscription with the present number, at $3·00 per annum.

By enlarging the Register we hope to lay before our readers, hereafter, every thing of real interest to the profession; providing more space for original matter, and, if possible, thus bring out some of the *hidden* talent. In other departments, also, we hope to present a more full *expose* of the advance of Dental Science. JAS. TAYLOR.

